# Preparation and optimization of aloe ferox gel loaded with Finasteride-Oregano oil nanocubosomes for treatment of alopecia

**DOI:** 10.1080/10717544.2022.2026534

**Published:** 2022-01-12

**Authors:** Khaled M. Hosny, Waleed Y. Rizg, Eman Alfayez, Samar S. Elgebaly, Abdulmohsin J. Alamoudi, Raed I. Felimban, Hossam H. Tayeb, Rayan Y. Mushtaq, Awaji Y. Safhi, Majed Alharbi, Alshaimaa M. Almehmady

**Affiliations:** a Department of Pharmaceutics, Faculty of Pharmacy, King Abdulaziz University, Jeddah, Saudi Arabia; b Center of Excellence for Drug Research and Pharmaceutical Industries, King Abdulaziz University, Jeddah, Saudi Arabia; c Department of Oral Biology, Faculty of Dentistry, King Abdulaziz University, Jeddah, Saudi Arabia; d Department of Clinical Biochemistry, Cairo Laboratories for clinical pathology, Cairo, Egypt; e Department of Pharmacology and toxicology, Faculty of Pharmacy, King Abdulaziz University, Saudi Arabia; f Department of Medical Laboratory Technology, Faculty of Applied Medical Sciences, King Abdulaziz University, Jeddah, Saudi Arabia; g Center of Innovation in Personalized Medicine (CIPM), 3D Bioprinting Unit, King Abdulaziz University, Jeddah, Saudi Arabia; h Center of Innovation in Personalized Medicine (CIPM), Nanomedicine Unit, King Abdulaziz University, Jeddah, Saudi Arabia; i Department of Pharmaceutics, College of Clinical Pharmacy, Imam Abdulrahman Bin Faisal University, Dammam, Saudi Arabia; j Department of Pharmaceutics, Faculty of Pharmacy, Jazan University, Jazan, Saudi Arabia; k Department of Pharmaceutical Chemistry, Faculty of Pharmacy, King Abdulaziz University, Saudi Arabia

**Keywords:** Finasteride, nanocubosomes, oregano oil, phytantriol, aloe ferox

## Abstract

Alopecia areata is a skin disorder characterized by scarless, localized hair loss that is usually managed by topical treatments that might further worsen the condition. Therefore, the current study aimed to develop nano-cubosomes loaded with finasteride (FI) and oregano oil (Or) to improve drug solubility and permeation through skin and then incorporate it into an aloe ferox gel base. An l-optimal coordinate exchange design was adopted to optimize nano-cubosomes. Phytantriol and Alkyl Acrylate were employed as the lipid material, and surfactant respectively for cubosomes manufacture. The produced formulations were assessed for their particle size, entrapment efficiency (EE%), FI steady-state flux (Jss) and minimum inhibitory concentration (MIC) against Pro-pionibacterium acnes. Optimal FI-Or-NCu had a particle size of 135 nm, EE% equals 70%, Jss of 1.85 μg/cm2.h, and MIC of 0.44 μg/ml. The optimum formulation loaded gel gained the highest drug release percent and ex vivo skin permeation compared to FI aqueous suspension, and pure FI loaded gel. Aloe ferox and oregano oil in the optimized gel formulation had a synergistic activity on the FI permeation across the skin and against the growth of p. acne bacteria which could favor their use in treating alopecia. Thus, this investigation affirms the ability of FI-Or-NCu loaded aloe ferox gel could be an effective strategy that would enhance FI release and permeation through skin and maximize its favorable effects in treating alopecia.

## 1. Introduction

Alopecia is a common disorder that results in hair loss in one or more areas of the body. This condition can manifest in a variety of ways depending on the severity and area affected, ranging from isolated or multiple small patches (Alopecia areata) to a diffuse hair loss on the scalp (Alopecia totalis) or on the entire body skin (Alopecia universalis) (Alopecia universalis) (Amin & Sachdeva, 2013; Safavi et al., 1995). Any hair-bearing area could be impacted by Alopecia, but the scalp is the most prominent part. Alopecia affects 2% of population with no perceivable difference between men and women (Lee et al., 2020). Despite the fact that the underlying causes of Alopecia remain an unknown, several studies have suggested that environmental, immunologic, and genetic factors may play a role in its progress (Darwin et al., 2018). Furthermore, the relationship between the microbial population that inhabits the scalp and hair growth abnormalities such as Alopecia areata (AA) has recently been the focus of attention among researchers and clinicians (Constantinou et al., 2021). It has recently been established that the bacteria *Propionibacterium acnes* is involved in the pathogenesis of AA (Wang et al., 2012).

5α-reductase enzyme in the skin, liver, prostate gland, and scalp converts testosterone to di-hydrotestosterone (DHT). There are two known isoforms of 5α-reductase, type I and type II. Type I is more prevalent in the skin, whereas type II is more prevalent in the urogenital tract and the internal root sheath of the hair follicle (Barquero-Orias et al., 2021). Evidence have shown that type II 5α-reductase has activity in hair follicle dermal papilla cell (Hoffmann & Happle, 1999). Moreover, there is evidence demonstrates that hair loss in men is associated with DHT-mediated effects on the hair follicle as men with hereditary deficit in type II 5α-reductase did not develop baldness (Andersson et al., 1991).

Finasteride is a 5α-reductase inhibitor which inhibits the conversion of testosterone to (DHT). It works more prevalently in type II isoform of 5α-reductase. By inhibiting this conversion, it lowers the DHT level significantly in the circulation and scalp. Moreover, oral finasteride dose can reduce the DHT levels by 65% in men with hair loss (Dallob et al., 1994). Upon oral administration, finasteride is rapidly absorbed and reaches a peak plasma concentration (C_max_) of 9.2 μg/L in 1 to 2 hours (t_max_) (McClellan & Markham, 1999). Finasteride has low solubility in water (11.7 mg/L) and low molecular weight 372.5 Da (Log *p* = 3) (Karrer-Voegeli et al., 2009). Moreover, finasteride penetration through skin layer can be a challenge. Soleymani et al has shown that *in vitro* skin permeability of finasteride can be enhanced through microemulsion formulation (Soleymani & Salimi, 2019).

Origanum vulgare, popularly known as wild marjoram (family Lamiaceae), has a long history of usage as a food and medicine and is native to the Mediterranean region (Cittera et al., 2000). Oregano oil is an essential volatile oil extracted from Origanum vulgare leaves. It contains various phenolic active constituents such as thymol and carvacrol (Yan et al., 2019). It is known as generally safe by FDA. It has grown interest for its various activities such as antioxidant (Yan et al., 2016), anti-inflammatory (Ocaña-Fuentes et al., 2010), anti-allergic (Lu et al., 2018). Studies have shown that its antimicrobial activity demonstrated in both *in-vitro* cell culture (Lopez-Reyes et al., 2010) and *in-vivo* systemic infections (Soylu et al., 2006).Aloe verox gel can be used in skin care products. It is known for restoring hydration to the skin, anti-inflammatory, anti-microbial, anti-erythema effects and hair growth effects. as a natural skin permeation enhancer. There are 400 species of Aloe belongings to the family Xanthorrhoeaceae (Fox et al., 2015). Drug permeation enhancers made from natural products are attracting considerable interest (Fox et al., 2011).

Nanocubosomes, which are cubic phase of liquid crystalline aggregates, possess a variety of characteristics that make them a broadly known drug delivery sysem. This kind of nano carriers are developed by amphiphilic lipids such as glycerol monooleate (GMO) and phytantriol (PHYT), which may self-assemble in water to form nanocubosomes (Pan et al., 2013). One of the key advantages of nanocubosomes as a promising delivery method is their potential to enhance the bioavailability of drugs that are poorly soluble in water. Furthermore, amphiphilic lipids (GMO) serve as a permeation enhancer while nanocubosomes penetrate skin layers (Ali et al., 2017).

In this wok, we aimed to Enhancee the solubility and permeation of Finasteride through the formulation of the drug in the form of nanocubosomes using Design of Experiments (DoE) to develop the best optimized formulation of the delivery vehicle. In this study, oregano oil was used as an oil component in the formulation of nanocubosomes while also taking advantage of its benefits in promoting hair growth, treating some scalp problems such as Alopecia Areata, and having antimicrobial activity that will stop the Propionibacterium acnes, which is the main bacterial infection that affects the scalp in cases of Alopecia Areata. Furthermore, Aloe ferox gel was used as a carrier for the loaded nanocubosomes to ensure intimate contact between the loaded nanocubosomes and the scalpel due to its adhesiveness and viscosity. Aloe ferox also has several benefits in skin treatment as an antimicrobial, anti-inflammatory, and hair growth promoter.

## 2. Materials and methods

### 2.1. Materials

Finasteride (FI) was obtained from Sigma-Aldrich Co. (St. Louis, MO, USA). Phytantriol was purchased from Avanti Polar Lipids (Alabaster, AL, USA). Alkyl Acrylate was attained from Lotioncrafter (Eastsound, Washington, USA). Sodium metabisulphite, and Benzalkonium chloride, were purchased from the BASF SE Chemicals Company (Ludwigshafen, Germany), Oregano oil was taken from Avanti Polar Liquids (AL, USA). Aloe Ferox extract was obtained from Beutsway Commercial foundation (Jeddah, Saudi Arabia).

### 2.2. Experimental design

In this work, an I-optimal design was performed by using various amounts of different materials, including (A) phytantriol, (B) Alkyl Acrylate, and (C) Oregano Oil. These materials are considered as independent factors. In the meantime, several dependent response parameters were performed, including the mean globule size of the formulated FI-Or-NCu (Y1), % entrapment efficiency (EE%) of the performed FI-Or-NCu loaded with FI (Y2), the FI steady-state flux throw the rat abdominal membrane (Y3), and MIC against Propionibacterium acnes (Prop. Acne) bacteria (Y4) (Table 1). All of the independent factor levels were chosen based on the results of the pre-optimization experiments. Each run was employed 50 mg of FI by the design. The EE can be used to calculate the weight of the prepared formulation that corresponds to the actual dosage of FI. The data was input once the FI-Or-NCu were produced according to formulas given by a design specialist.

**Table 1.** The coded values for the independent factors and the results of dependent responses for the different formulations of FI-Or-NCu.

	Factor A	Factor B	Factor C	Response 1	Response 2	Response 3	Response 4	
Run	Phytantriol (mg)	Alkyl Acrylate (mg)	Oregano Oil (mg)	Particle size (nm)	EE (%)	Jss (µg/cm2.h)	MIC (µg/ml)	PDI
1	0.18	−1	−0.32	145	81	1.73	0.681	0.12
2	0.17	−1	1	150	80	2.71	0.134	0.10
3	−1	0.19	1	92	55	2.84	0.211	0.13
4	1	−0.3	0.3	170	93	2.09	0.491	0.11
5	1	−0.3	0.3	155	92	2.1	0.399	0.16
6	−0.3	1	0.3	140	64	2.4	0.399	0.21
7	−1	0.27	−0.22	95	54	1.81	0.599	0.28
8	−0.3	−0.3	−1	115	71	1.21	0.733	0.19
9	−1	−1	−0.177496	89	57	1.91	0.587	0.26
10	0.95	0	−0.71	165	89	1.61	0.644	0.22
11	0	0	0	121	77	2.04	0.498	0.18
12	−1	1	−1	116	52	1.42	0.745	0.33
13	1	−1	−1	160	96	1.11	0.762	0.31
14	−0.3	1	0.3	131	65	2.39	0.296	0.27
15	1	1	−1	164	87	1.53	0.646	0.34
16	−0.00975064	0.937024	−0.69961	140	73	1.69	0.688	0.24
17	1	1	1	185	88	2.89	0.273	0.35
18	−0.3	−0.3	−1	129	72	1.34	0.751	0.30
19	0.110859	0.1	−0.1	146	79	1.96	0.536	0.29
20	0.331759	0.164936	1	160	83	2.79	0.221	0.14

### 2.3. Fi-or-NCu preparation

Several FI-Or-NCu formulations were made using various concentrations of phytantriol (750 to 1250 mg), alkyl acrylate (150 to 450 mg), and Oregano oil (25–75 mg) according to the proposed design. Phytantriol was weighed accurately in specific amounts. Then, it was heated at 45 °C in a glass in order to produce a free-flowing powder. After that, specific amounts of oregano oil was weighed accurately and added to the prepared powder. Then, they were mixed well in order to produce a creamy dispersion. Subsequently, a specific amount of FI (50 mg) and specific alkyl acrylate were added to cubosome glass. The powders were dissolved in 10 mL buffer that contains a pH of 7.4. Then, the obtained mixture was homogenized and conducted out at 45 °C and 14,000 rpm for 10 min in order to achieve a milky dispersion. The performed dispersions were collected and then stored at 4 °C for further evaluation (Chang et al., 2021).

### 2.4. Evaluation of prepared FI-or-NCu different formulations

#### 2.4.1. Particle size of FI-or-NCu

After diluting the FI-Or-NCu dispersion 20 times with the PBS buffer that contains a pH of 7.4, the particle size for the produced FI-Or-NCu preparations were measured in triplicate using a Microtrac® zetatrack particle size analyzer.

#### 2.4.2. Fi entrapment efficiency percentage

In the beginning, the prepared FI-Or-NCu dispersions were centrifuged at 10,000 rpm for 30 min at 4 °C to estimate the EE% of FI. The resultant supernatant was removed in order to isolate the cubosome pellet, which was subsequently sonicated in methanol (15 min). Finally, the FI content was calculated using high-performance liquid chromatography (HPLC) throw chromatographic separation. The flow rate was performed at 1 mL/min. A phenomenex C18 column (150 mm x 4.6 mm, 5.0 particle size) was used with a mobile phase containing 0.02% formic acid (in water) and methanol in a 1:4 (v/v) ratio. The FI was eluted at 3.4 min. Equation (1) was used to compute the EE%, where FI1 represents the entire amount of entrapped FI and FI2 represents the total amount of unentrapped FI. The EE% is the percentage of total FI encapsulated in the FI-Or-NCu produced formulation.

(1) EE% = [FI1 – FI2]/FI1 * 100……

#### 2.4.3. *Ex vivo* skin permeation of FI-or-NCu

In this study, the animal models were obtained from Cairo Agriculture for Experimental Animals, Cairo, Egypt. Moreover, the animals were utilized with the approval of the Research Ethics Committee of the CAE Center for Clinical Laboratories in Cairo, Egypt, with the guarantee of following the Helsinki Agreement procedures and the Guiding Principles in Animal Care and Use (DHEW published NIH 80–23) (Approval No (292-07-21). Microette Plus Hanson Automated Vertical Diffusion Cells (Hanson Research, Chatsworth, CA, USA) was conducted for performing *ex-vivo* skin permeation studies. The PBS with a pH of 5.8 was used as the diffusion medium. The medium was maintained at 37 °C ± 0.5 °C with continuous stirring (400 rpm). Male Wistar rats were used and their skins were excised from abdominal regions. Then, the excised skins were cleaned, examined for integrity and prepared for investigation by soaking PBS (pH 5.8) for 3 h. After that, the treated skins were mounted between donor and receptor compartments of diffusion cells separately.

The HPLC was used to calculate the cumulative amount of FI permeated. The Phenomenex C18 column (150 mm x 4.6 mm, 5.0 µ particle size) was used with a mobile phase formic acid (0.02%) (in water) and methanol in the ratio of 1:4 (v/v). The flow rate of the mobile phase was performed at 1 mL/min, and the FI was eluted at 3.4 min. The steady-state flux (Jss) for each sample was calculated using the slope of the curve plot between the cumulative percentage penetrated and time (Hosny et al., 2021).

#### 2.4.4. Assessment of MIC of the prepared FI-or-NCu against prop. Acnes

In the beginning, an agar dilution method was utilized in order to determine the minimal inhibitory concentration (MIC) values (Lamlertthon et al., 2007). To obtain the appropriate concentration range of tested material (0.01–3.0% V/V), different dispersions of FI-Or-NCu were introduced aseptically to 10 mL aliquots of sterile molten agar containing 0.5% tween 80. Then, the obtained solutions were vortexed for 30 seconds or until completely dispersed. Then, the solution was immediately poured into sterile Petri dishes then allowed to be placed for 60 min. The organisms that will be tested P. acne (1 L) from the prepared inoculum was added to the plates, which were allowed until the inoculum had set before being incubated at 37 °C for 72 h under anaerobic conditions as stated before. Subsequently, the utilized plates were observed and recorded for the presence or absence of growth. The results showed that the MIC was noted as the lowest concentration of test materials where the absence of growth was observed.

### *2.5. Optimization of* FI-or-NCu

2.5

The formulation of FI-Or-NCu was optimized under the restrictions and goals that listed in Table 2. The outcomes of the formulation experiments were statistically analyzed, and numerical optimization of the FI-Or-NCu formulation was done to meet the set targets in terms of responses. Then, the optimized FI-Or-NCu formulations were assessed for different parameters, including globule size, MIC, Jss, and EE% as mentioned previously. The improved FI-Or-NCu formulation was then added into the aloe ferox gel basis, and the loaded gel was made and analyzed.

**Table 2.** Constraints and goals selected to optimize the FI-Or-NCu formulation.

Factor	Name	Constrains	Optimum Value
Independent factors	Phytantriol (mg)	750–1250 mg	992 mg
	Alkyl Acrylate (mg)	150–450 mg	450 mg
	Oregano Oil (mg)	25–75 mg	38 mg

### 2.6. Preparation of aloe ferox gel loaded with Finasteride-Oregano oil nanocubosomes (FI-or-NCu)

In the beginning, several ingredients were accurately weighed separately for preparing the aloe ferox gel, including, sodium metabisulphite (0.5%,), and benzalkonium chloride (0.1%). After that, the ingredients were dissolved in distilled water. Then, an accurately weighed Hydroxypropyl cellulose (1%) was added to the prepared solution. Then, the mixture was continuously stirred until it was completely swollen. A precise amount of FI-Or-NCu was introduced into the swelled mixture with continuous stirring to make 5% of FI. This stiff gel-like mixture was then added to the aloe ferox extract (50 mL) and mixed for 15 min. With the addition of water and continual stirring, a suitable volume was attained, and a homogenous gel was created.

### 2.7. Evaluation of gel

#### 2.7.3. *In vitro* release of the FI-or-NCu nanocubosomes -loaded aloe ferox gel

The *In vitro* drug release study was done for various preparations, including 1) FI 5% aloe ferox gel that contains the optimized FI-Or-NCu [Gel 1]; 2) FI 5% aloe ferox gel that made by using pure powder of FI as an alternative of FI-Or-NCu; [Gel 2]; 3) aqueous dispersion of FI 5% [Susp] in this method, a USP dissolution apparatus (Type I, basket type; DT 700 LH device, Erweka GmbH DT 700, Heusenstamm, Germany) was performed. In the beginning, the prepared formulations were set into separate cylindrical tubes, which has a diameter of 2.7 cm and a length of 10 cm. The utilized buffer was maintained at 37 ± 0.5 °C and it was continuously stirring at 50 rpm. This dissolution test was performed for 3 h. Then, several samples were collected at various time intervals. Then, the withdrawal samples were replaced carefully with the same amount of dissolution medium.

#### 2.7.4. *Ex vivo* skin permeation of aloe ferox gel loaded with FI-or-NCu nanocubosomes

In this method, the *Ex vivo* skin permeation study was performed for the prepared aloe ferox gel that loaded with FI-Or-NCu formulation following the above-stated procedure. Different formulations were tested. The first formulation is the FI 5% aloe ferox gel that is incorporated with FI-Or-NCu [Gel 1]. The second formulation is FI 5% ferox gel prepared. This gel was prepared by using FI powder instead of FI-Or-NCu; [Gel 2]. The third formulation is FI 5% aqueous dispersion. [Susp.]. At each sampling period, the proportion of FI penetrated was calculated, and the percentage of FI permeated and the steady state flux (Jss) for each sample were acquired.

#### 2.7.5. Assessment of MIC of the prepared aloe ferox gel loaded with FI-or-NCu nanocubosomes against prop. Acnes

Antibacterial activity was tested on a variety of formulations, including the FI 5% aloe ferox gel loaded with FI-Or-NCu [Gel 1], the formulated FI 5% aloe ferox gel which was formulated by using pure FI powder instead of FI-Or-NCu [Gel 2], the pure aloe ferox gel that is incorporated with Or-NCu without using FI [Gel 3], the FI 5% aloe ferox gel loaded with a mixture of FI and Or that not entrapped within NCu). [ Gel 4], and the FI-Or-NCu 5% aqueous dispersion that done by utilizing distilled water as a replacement for aloe ferox extract [Susp].

#### 2.7.6. Statistical analysis

The data was presented as a mean and standard deviation. The paired t-test was used to make statistical comparisons between the outcomes of the different groups (*p* < .05 was considered significant).

## 3. Results and discussion

### 3.1. Experimental design analysis

The employed experimental design, which was l-optimal coordinate exchange design was developed by the analytical software Design Expert ® to test the model fit and adequacy, determine the design parameter and investigate the effect of each factor or its interactions on the dependent responses. The selected model’s validity was tested using ANOVA analysis, correlation coefficients at a meaning rate of 95 percent (*p* < .05), and F values. Further, the precision and accuracy of the obtained mathematical model for measured responses’ predictions was confirmed by a check point analysis. The response surface plots, and main effect diagrams were developed, in addition to the overlaying region of the desired response corresponding to the optimum region where the most desired nano cubic dispersion can be obtained. Finally, desirability values were allocated, and the actual and predicted characteristics of optimal formulation were compared.

### 3.2. Characterization of FI-or-NCu

#### 3.2.1. Particle size measurements

As presented in Table 2, the average diameter of FI-Or-NCu vesicles ranged between 89 and 185 nm with polydispersity index values between 0.10 to 0.35, which might be deemed as a respectable mid-range (Lamlertthon et al., 2007). Such results showed a satisfactory size distribution and acceptable formulation homogeneity. The vesicles’ size values were capitulated to using a linear model of polynomial analysis with *p*-value < .0001 and lack of fit *p*-value equals .5345. L-optimal statistical analysis illustrated the developed model’s ability to investigate the significant effect of phytantriol (A), Alkyl Acrylate (B), and Oregano Oil (C)on the particle size of the nano-vesicles. The obtained model gained an experimental R2 of 0.8763, which was very close to the expected R2 (i.e. 0.8191), as seen in Table 3. ANOVA analysis of the gathered data yielded the equation below.

(3) Particle size= +135.66+34.46 A+6.20 B+3.49 C

**Table 3.** Regression analysis results for Y1, Y2, Y3, and Y4 responses.

Dependent variables	R2	Adjusted R2	Predicted R2	F-value	p-value	Adequate precision
Y1	0.8958	0.8763	0.8314	45.87	0.0001	20.0412
Y2	0.9946	0.9897	0.9674	204.85	0.0001	43.4217
Y3	0.9913	0.9872	0.9774	245.76	0.0001	47.8561
Y4	0.9301	0.9170	0.8789	71.02	0.0001	72.4208

**Figure 1.**
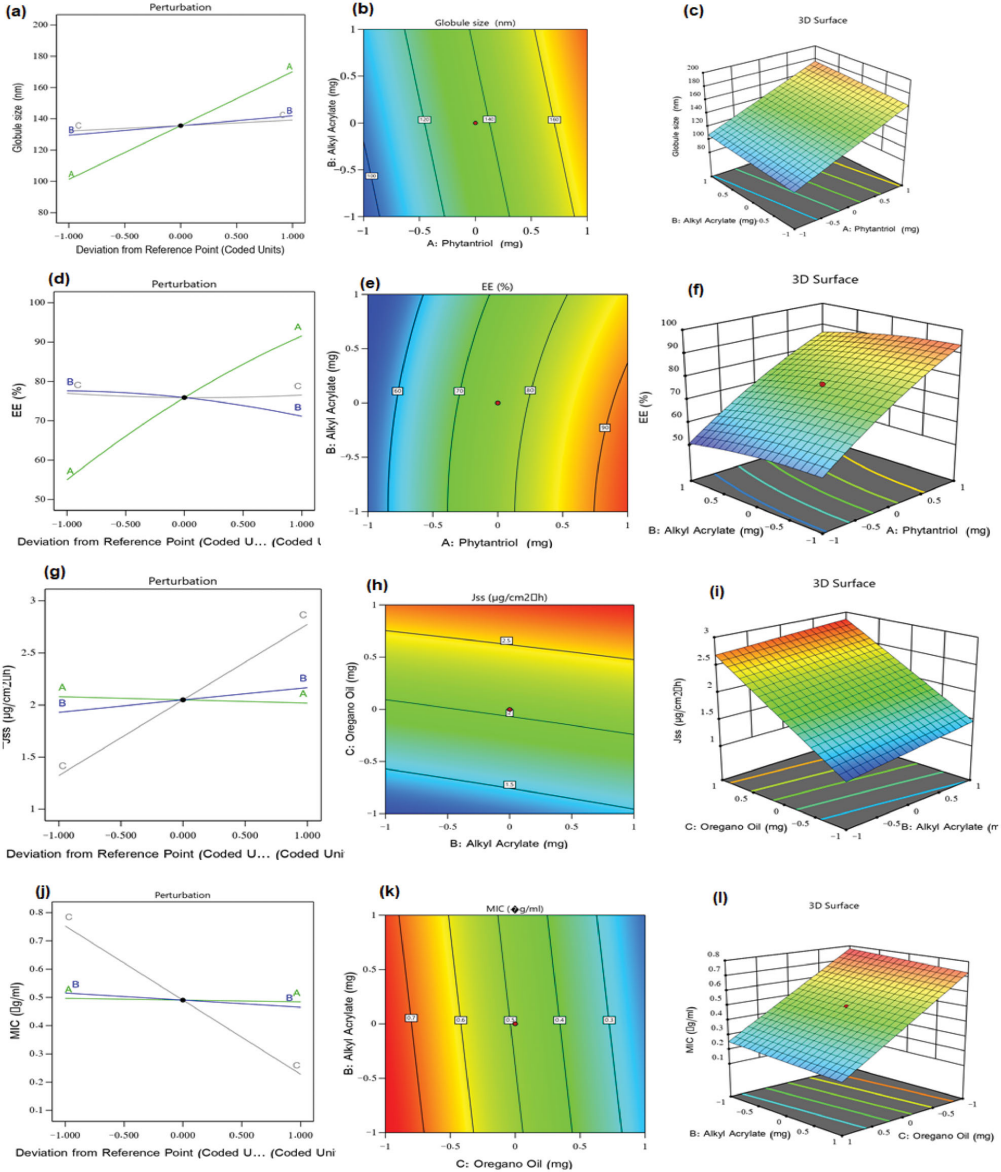
Design plots of the various responses of FI-Or-NCu formulations: (a) perturbation plot for particle size, (b) contour plot for particle size, (c) response surface plot for particle size, (d) perturbation plot for EE%, (e) contour plot for EE%, (f) response surface plot for EE%, (g) perturbation plot for Jss, (h) contour plot for Jss, (i) response surface plot for Jss, (j) perturbation plot for MIC, (k) contour plot for MIC, and (l) response surface plot for MIC.

As could be noticed from perturbation diagram, contour, and 3 D-surface plot of particle size response (Figure 1), all of the investigated factors acquired a positive effect on particle size, however, phytantriol (factor A) exerted the most prominent effect on the developed vesicles’ size. This result can be also confirmed by the high coefficient value of factor A observed in the developed model’s equation. The larger vesicles obtained by increasing phytantriol amount could be ascribed to the lower shearing effects and higher possibility of vesicles aggregation at higher phytantriol levels, similar results were reported in literature (Alharbi & Hosny, 2020).

#### 3.2.2. Entrapment efficiency (EE %) determination

Data collected in Table 2 revealed that the mean EE% of FI in FI-Or-NCu vesicles fluctuated between 52 and 96%. The used software suggested a quadratic surface response for EE% values evaluation. The proposed model acquired a *p*-value <.0001 and lack of fit *p*-value of .1017. Therefore, the developed model was able to examine the effect of phytantriol (A), Alkyl Acrylate (B), and Oregano Oil (C) on the EE% of the drug into the nano-vesicles. The selected model had comparable adjusted and predicted R2 values of 0.9897 and 0.9674 respectively, as observed in Table 3. Analysis of variance the obtained data resulted in the following equation.

(4) EE%=+75.88 +18.28A−3.24B−0.2220C−0.3442AB +0.3943AC+0.4145BC−2.62A2−1.50B2+0.8772C2

These outcomes illustrated the superior effect of phytantriol on EE%. Upon examining the perturbation diagram, contour, and 3 D-surface plot of EE% response (Figure 1), it was found that phytantriol level had the most paramount effect on EE% as higher phytantriol levels would encourage a great increase in EE%, similar findings were observed in ciprofloxacin loaded phytantriol-based cubosomes (Alharbi & Hosny, 2020). Such result could be referred to the boosted rigidity of the vesicles’ lipid bilayer at higher phytantriol levels, in addition to feasible formation of cubosomes with multilayered membranes, and the superior affinity of lipophilic drugs toward phytantriol (El-Nabarawi et al., 2013; Perugini & Pavanetto, 1998). Interestingly, Alkyl Acrylate was found to have an antagonistic effect on EE % of FI which might be explained considering the high molecular weight of Alkyl Acrylate, which could disrupt the integrity of the cubosomes’ lipid bilayer leading to drug diffusion into the surrounding aqueous medium during the step of isolating free drug from the drug-laded vesicles (Salama et al., 2012; Shelke et al., 2016).

#### 3.2.3. *Ex vivo* skin permeation study of FI-or-NCu

Steady state flux of FI of the investigated cubosomes through rat skin acquired values between 1.11 and 2.89 µg /cm2.h, as summarized in Table 2.

The highest significant average squared value exceeding the residual error (*p* ˂.0002) was achieved by a 2FI model of polynomial analysis, therefore, it was used for analysis of FI Jss data. The adopted statistical design revealed the mathematical model’s ability to assess the significant effect of phytantriol (A), Alkyl Acrylate (B), and Oregano Oil (C) on FI Jss of FI-Or-NCu. The determined model gained an adjusted R2 value of 0.9872, which was in line with a predicted R2 of 0.9774, Table 3. ANOVA analysis of the obtained data revealed the following equation:

(5) Jss=+2.05 −0.0310A+0.1187B+0.7261C+0.0784AB−0.0451AC−0.0283BC

Notably, the perturbation diagram, contour, and 3 D-surface plot of drug Jss response (Figure 1), revealed that oregano oil level had the most significant effect on Jss as increasing oregano oil levels (factor C) would highly facilitate drug permeation across rat skin. These findings can be understood in the light of oregano oil chemical composition. Carvacrol, which is the major component of oregano oil, was well known as a skin permeation enhancer that could efficiently deliver FI through the excised rat skin owing to its hydrogen-binding ability via the hydroxyl group and aromaticity (Songkro et al., 2009), hence, it could disorganize the close packing pattern of intercellular lipid matrix of skin layers (Williams & Barry, 2004). Besides, the amount of alkyl acrylate (factor B) had a positive effect on drug permeation across skins, as the increase in its amount caused a slight increase in FI penetration across skin. Such effect could be due to the emulsifying and penetration enhancing nature of alkyl acrylate which could have integrated with skin lipids to form channels that would facilitate drug permeation, comparable results were mentioned in literature (Kizeviciene et al., 2017; Shin et al., 2001).

#### 3.2.4. Assessment of MIC of the prepared FI-or-NCu against P. acnes

MIC of the produced cubosomes against P. acnes oscillated between 0.134 and 0.762 µg /ml, as summarized in Table 2.

The adopted design of experiments detected the independent variables’ effect on MIC values of FI-Or-NCu. The most significant average squared value surpassing the residual error (*p* ˂ .0001) was acquired by a linear model of polynomial analysis, consequently, it was used to test MIC data. The allocated model procured a predicted R2 value of 0.8789, which was in line with an adjusted R2 of 0.9170, Table 3. ANOVA analysis of obtained data revealed the following equation:

(6) MIC=+0.4904 −0.0062A−0.0254B−0.2626C

Upon exploring Equation 6 along with the perturbation, contour, and 3 D-surface plots, it appeared that all the tested factors exerted an antagonistic effect on MIC, however, factor C (i.e. oregano oil amount) attained the most important impact on the response 4. Thus, increasing oregano oil amount would result in a great decrease in MIC values.

The major chemical constituents of oregano oil (i.e. terpenoids) basically target the bacterial cell membrane, as they could disrupt with its phospholipid bilayers (Gumus et al., 2010). Thymol and carvacrol might decompose the microbial cell membrane, hence release the internal lipopolysaccharide compounds, increase the adenosine triphosphate diffusion, and collectively change the cellular passive permeability (Guarda et al., 2011). The great capacity of OEO to prevent the growth of varying bacterial strains was reported by several research groups (Cosge et al., 2009).

It was also observed that the increase in factors A and B amounts led to a slight decrease in MIC values. Alkyl Acrylate, as a polymeric emulsifier could have increased the fluidity of bacterial cell membrane leading to diffusion of bacterial cell components, and eventually led to cell death, similar outcomes were previously reported (Hunter et al., 1995). Phytantriol also exerted an inhibitory effect on p.acne growth which could be explained by its great cytotoxic effect exerted by damaging the bacterial cell was as formerly reported by other researchers (Hunter et al., 1995; Hinton et al., 2014).

### 3.3. Optimization of FI-or-NCu

**Figure 2.**
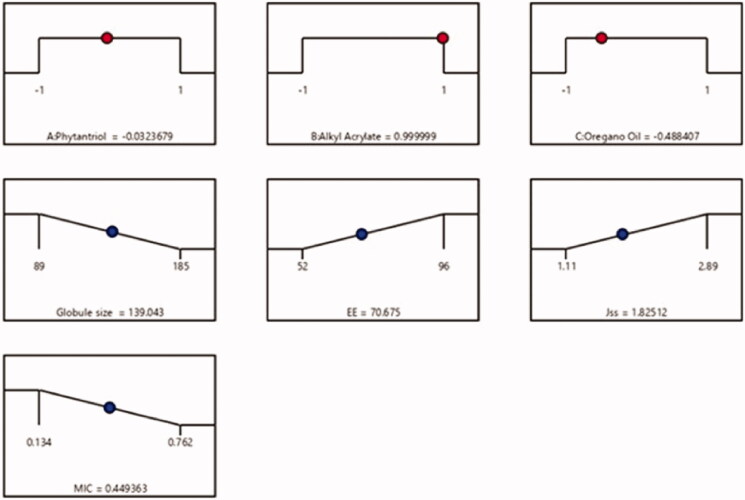
Desirability ramp showing the levels of independent variables and predicted values for the responses of the optimum formulation.

Utilizing the preceding data, an optimized FI-Or-NCu formulation was fabricated, enforcing the most appropriate characters. Diverse solutions were proposed by Design expert ® software applying several combinations of different levels of studied factors. The optimized formulation composed of 992, 450, and 38 mg of factors A, B and C, respectively. The obtained optimum cubosome formulation had a particle size of 135 nm, EE% of 70%, Jss of 1.85 µg /cm2.h, and MIC of 0.44 µg/ml with 0.845 desirability. Figure 2 presented the desirability ramp for varying levels of investigated factors and expected values of the dependent variables of the optimum formulation. Table 4 contained that the actual values of the optimal formulation’s responses which were in close agreement with predicted ones with no considerable variations (*p* > .05), proving the models’ validity and precision.

**Table 4.** Actual and experimental values of the optimized FI-Or-NCu formulation.

Solution	Phytantriol (mg)	Alkyl Acrylate (mg)	Oregano Oil (mg)	particle size (nm)	EE (%)	Jss (µg/cm2.h)	MIC (µg/ml)	Desirability
Predicated value	922	450	38	139	70.6	1.82	0.449	0.845
Experimental value	922	450	38	135	70	1.90	0.438	0.845

#### 3.3.1. Check-point analysis

Predicted and adjusted R2 values proved the oracular accuracy of the suggested regression models. Moreover, the actual to expected values ratios attained low percentage error and acceptable residuals between the expected and actual responses, recommending the lack of curvature in the data and validity of the model, as shown in Table 5.

**Table 5.** Composition and % prediction error between actual and expected responses of the optimal FI-Or-NCu formulation.

Factor	Optimal value	Response Variable	% Prediction error^a^
A: Phytantriol (mg)	992	Particle size (nm)	−2.9 %
B: Alkyl Acrylate (mg)	450	EE (%)	−0.85%
C: Oregano oil (mg)	38	Jss (µg/cm2.h)	3%
		MIC (µg/ml)	−0.9%

^a^Calculated as [Actual-predicted/Actual] *100.

### 3.4. *In vitro* release of FI from the FI-or-NCu nanocubosomes -loaded aloe ferox gel

**Figure 3.**
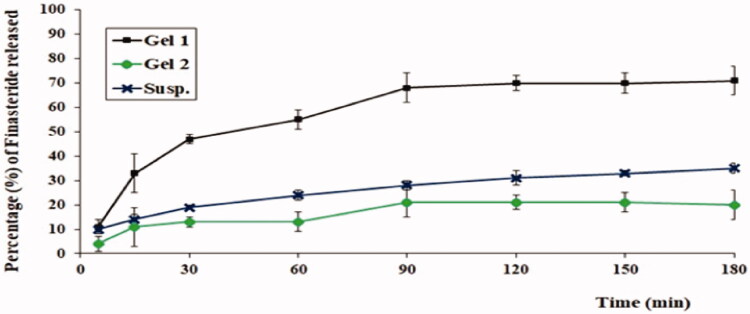
The in-vitro release profiles of FI from different formulations.

As could be noticed from Figure 3, aloe ferox gel loaded with optimized FI-Or-NCu (Gel 1) exhibited the highest release percentage of FI followed by FI aqueous suspension, while aloe ferox gel prepared by using pure FI powder attained the lowest release % of drug. Such findings can be explained considering the cubosomes size as it falls in the nano-range hence offers a large surface area for drug release, moreover, drug is present in the solubilized form in the lipid membrane of the nano-cubosomes which facilitates is its dissolution and release. On the other hand, the pure drug contained in gel matrix will suffer from slow diffusion due to the high viscosity of the gel which would hinder its release (Salem et al., 2021).

### 3.5. *Ex vivo* skin permeation of aloe ferox gel loaded with FI-or-NCu

**Figure 4.**
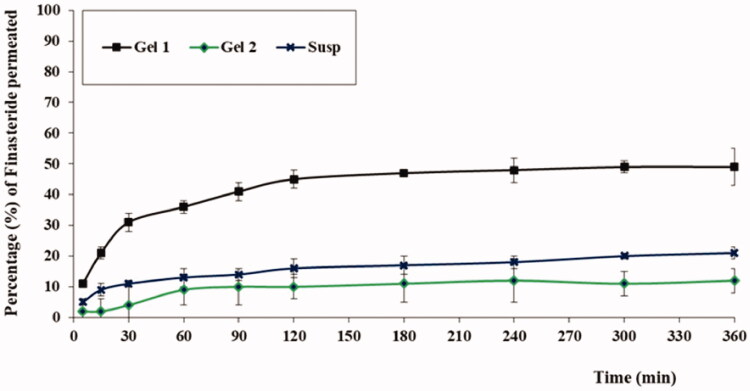
The ex-vivo permeation profiles of FI from different formulations.

Figure 4 presented the permeation profiles of FI from the different tested formulations. As could be observed, aloe ferox gel loaded with optimized FI-Or-NCu (Gel 1) exhibited the highest permeation percentage of FI followed by FI aqueous suspension, while aloe ferox gel prepared by using pure FI powder attained the lowest permeation % of FI. The steady state flux for the tested formulations calculated from the slope of the graph was 1.4 µg/cm^2^.h for (Gel 1) compared to 0.31 µg/cm^2^.h for aqueous dispersion of FI. The high permeation % achieved by the gel base loaded with FI-Or-NCu could be attributed to the penetration enhancing properties of cubosomes. As previously discussed, the components of developed vesicles acquired permeation enhancing properties. Carvacrol, the major component of oregano oil, is the main reason behind the skin permeation enhancing properties of Or oil owing to its hydrogen-binding ability via the hydroxyl group and aromaticity (Songkro et al., 2009), hence, it could disorganize the close packing pattern of intercellular lipid matrix of skin layers (Williams & Barry, 2004). Moreover, alkyl acrylate could improve drug permeation across skin due to its emulsifying and penetration enhancing nature which could be due to its integration with skin lipids to form channels that would facilitate drug permeation, comparable results were mentioned in literature (Kizeviciene et al., 2017; Shin et al., 2001).

**Figure 5.**
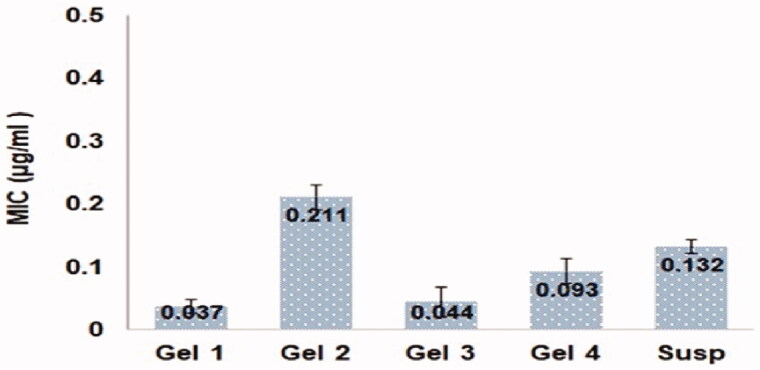
MIC values for different FI loaded formulations: (1) 5% FI aloe ferox gel loaded with FI-Or-NCu [Gel 1], (2) 5% FI aloe ferox gel loaded with pure FI powder [Gel 2], (3) plain aloe ferox gel loaded with Or-NCu without FI [Gel 3], (4) 5% FI aloe ferox gel loaded with mixture of Or and FI (not entrapped within NCu) [Gel 4], and (5) 5% FI-Or-NCu aqueous dispersion prepared by using distilled water instead of aloe ferox extract [Susp].

### 3.6. Assessment of MIC of the prepared aloe ferox gel loaded with FI-or-NCu nanocubosomes against prop. Acnes

Figure 5 shows the MIC values for different FI loaded formulations, it was noticeable that the gel formulation loaded with FI-Or-NCu acquired the lowest MIC value (0.037 µg/ml), while the gel formulation loaded with pure drug attained the highest MIC value (0.211 µg/ml). The superior antimicrobial activity of gel base loaded with FI-Or-NCu could be referred to the chemical constituents of oregano oil (i.e. terpenoids) that largely target the bacterial cell membrane, and disrupt with its phospholipid bilayers (Gumus et al., 2010) releasing the internal lipopolysaccharide compounds, increasing the adenosine triphosphate diffusion, and finally change the cellular passive permeability (Guarda et al., 2011). The great capacity of oregano essential oil to prevent the growth of varying bacterial strains was reported by several research groups (Cosge et al., 2009). In addition, Alkyl Acrylate, as a polymeric emulsifier could have increased the fluidity of bacterial cell membrane leading to diffusion of bacterial cell components, leading to cell death. similarly, phytantriol also could inhibit p.acne growth due to its great cytotoxic effect exerted by damaging the bacterial cell, similar results were formerly reported (Hunter et al., 1995). Formulation gel 3 (i.e. contained cubosomes without FI) and formulation gel 4 (i.e. contained oregano oil and FI in a free form not entrapped in cubosomes) had MIC values that were higher than formulation gel 1 that contained FI-Or-NCu and lower than formulation gel 2 that contained pure FI. Such outcomes reveal the bacterial growth inhibitory properties of each component only, but more importantly it signifies the synergistic effect found between FI and oregano oil against the growth of p. acne bacteria which could favor their use in treating alopecia.

## 4. Conclusion

In the current study, l-optimal coordinate exchange design was adopted for optimization of FI-Or-NCu and the optimal formulation was successfully mixed with aloe ferox gel base. FI was effectively incorporated into cubosomes with adequate properties. The optimized formulation gained particle size, EE%, Jss, and MIC values of 135 nm, 70%, 1.85 µg/cm2.h, and 0.44 µg/ml respectively. Additionally, optimized formulation acquired enhanced in vitro drug release and ex vivo permeation compared to varying formulations. Finally, the optimum formulation showed the lowest MIC values among other tested formulations confirming its superior antimicrobial activity in addition to the synergistic antimicrobial effect of oregano oil and FI. Thus, this investigation affirms the ability of FI-Or-NCu loaded aloe ferox gel could be an effective strategy that would enhance FI release and permeation through skin and maximize its favorable effects in treating alopecia areata.

## Data Availability

Data used to support the findings of this study are included within the article.

## References

[CIT0001] Alharbi WS, Hosny KM. (2020). Development and optimization of ocular in situ gels loaded with ciprofloxacin cubic liquid crystalline nanoparticles. J Drug Deliv Sci Technol 57:101710.

[CIT0002] Ali MA, Kataoka N, Ranneh A-H, et al. (2017). Enhancing the solubility and oral bioavailability of poorly water-soluble drugs using monoolein cubosomes. Chem Pharm Bull 65:42–8. 10.1248/cpb.c16-00513 28049915

[CIT0003] Amin SS, Sachdeva S. Alopecia areata: a review. J Saudi Soc Dermatology Dermatologic Surg 2013;17:37–45.

[CIT0004] Andersson S, Berman DM, Jenkins EP, Russell DW. (1991). Deletion of steroid 5α-reductase 2 gene in male pseudohermaphroditism. Nature. 354:159–61. 1944596 10.1038/354159a0 PMC4451825

[CIT0005] Barquero-Orias D, Muñoz Moreno-Arrones O, Vañó-Galván S. (2021). Alopecia and the microbiome: a future therapeutic target? Actas Dermo-Sifiliográficas 112:495–502.

[CIT0006] Chang C, Meikle TG, Drummond CJ, et al. (2021). Comparison of cubosomes and liposomes for the encapsulation and delivery of curcumin. Soft Matter 17:3306–13. 33623948 10.1039/d0sm01655a

[CIT0007] Cittera A, Cazzola R, Cestaro B, Precliniche S. (2000). Antioxidant properties of oregano (Ohganum Vulgare) leaf extracts. Water 24:453–65.

[CIT0008] Constantinou A, Kanti V, Polak-Witka K, et al. (2021). The potential relevance of the microbiome to hair physiology and regeneration: the emerging role of metagenomics. Biomedicines 9:236. 33652789 10.3390/biomedicines9030236 PMC7996884

[CIT0009] Cosge B, Turker A, Ipek A, et al. (2009). Chemical compositions and antibacterial activities of the essential oils from aerial parts and corollas of Origanum acutidens (Hand.-Mazz.) Ietswaart, an endemic species to turkey. Molecules 14:1702–12. 19471191 10.3390/molecules14051702 PMC6254327

[CIT0010] Dallob AL, Sadick NS, Unger W, et al. (1994). The effect of finasteride, a 5 alpha-reductase inhibitor, on scalp skin testosterone and dihydrotestosterone concentrations in patients with male pattern baldness. J Clin Endocrinol Metab 79:703–6. 8077349 10.1210/jcem.79.3.8077349

[CIT0011] Darwin E, Hirt P, Fertig R, et al. (2018). Alopecia areata: review of epidemiology, clinical features, pathogenesis, and new treatment options. Int J Trichol 10:51–60. 10.4103/ijt.ijt_99_17 PMC5939003 29769777

[CIT0012] El-Nabarawi MA, Bendas ER, El Rehem RTA, Abary MYS. (2013). Transdermal drug delivery of paroxetine through lipid-vesicular formulation to augment its bioavailability. Int J Pharm 443:307–17. 23337629 10.1016/j.ijpharm.2013.01.016

[CIT0013] Fox LT, Gerber M, Du Plessis J, Hamman JH. (2011). Transdermal drug delivery enhancement by compounds of natural origin. Molecules 16:10507–40.

[CIT0014] Fox LT, Gerber M, Preez JLD, et al. (2015). Skin permeation enhancement effects of the gel and whole-leaf materials of Aloe vera, Aloe marlothii and Aloe ferox. J Pharm Pharmacol 67:96–106. 25196486 10.1111/jphp.12311

[CIT0015] Guarda A, Rubilar JF, Miltz J, Galotto MJ. (2011). The antimicrobial activity of microencapsulated thymol and carvacrol. Int J Food Microbiol 146:144–50. 21411168 10.1016/j.ijfoodmicro.2011.02.011

[CIT0016] Gumus T, Demirci A, Sagdic O, Arici M. (2010). Inhibition of heat resistant molds: Aspergillus fumigatus and Paecilomyces variotii by some plant essential oils. Food Sci Biotechnol 19:1241–4.

[CIT0017] Hinton TM, Grusche F, Acharya D, et al. (2014). Bicontinuous cubic phase nanoparticle lipid chemistry affects toxicity in cultured cells. Toxicol Res 3:11–22.

[CIT0018] Hoffmann R, Happle R. (1999). Finasteride is the main inhibitor of 5alpha-reductase activity in microdissected dermal papillae of human hair follicles . Arch Dermatol Res 291:100–3. 10195397 10.1007/s004030050390

[CIT0019] Hosny KM, Rizg WY, Alkhalidi HM, et al. (2021). Nanocubosomal based in situ gel loaded with natamycin for ocular fungal diseases: development, optimization, in-vitro, and in-vivo assessment. Drug Deliv 28:1836–48. 34515597 10.1080/10717544.2021.1965675 PMC8439233

[CIT0020] Hunter RL, Jagannath C, Tinkley A, et al. (1995). Enhancement of antibiotic susceptibility and suppression of Mycobacterium avium complex growth by poloxamer 331. Antimicrob Agents Chemother 39:435–9. 7726511 10.1128/aac.39.2.435 PMC162556

[CIT0021] Karrer-Voegeli S, Rey F, Reymond MJ, et al. (2009). Androgen dependence of hirsutism, acne, and alopecia in women: retrospective analysis of 228 patients investigated for hyperandrogenism). Medicine 88:32–45. 19352298 10.1097/md.0b013e3181946a2c

[CIT0022] Kizeviciene E, Jonaitiene L, Peciura R. (2017). Evaluation of acrylates/c10-30 alkyl acrylate cross- polymer mixture effectiveness on o/w type emulsion formulation. Acta Pol Pharm 74:937–43. 29513964

[CIT0023] Lamlertthon S, Luangnarumitchai S, Tiyaboonchai W. (2007). Antimicrobial activity of essentials oils against five strains of Propionibacterium acnes. Mahidol University J Pharma Sci 34:60–4.

[CIT0024] Lee HH, Gwillim E, Patel KR, et al. (2020). Epidemiology of alopecia areata, ophiasis, totalis, and universalis: a systematic review and meta-analysis. J Am Acad Dermatol 82:675–682. 31437543 10.1016/j.jaad.2019.08.032

[CIT0025] Lopez-Reyes JG, Spadaro D, Gullino ML, Garibaldi A. (2010). Efficacy of plant essential oils on postharvest control of rot caused by fungi on four cultivars of apples in vivo. Flavour Fragr J 25:171–7.

[CIT0026] Lu M, Dai T, Murray CK, Wu MX. (2018). Bactericidal property of oregano oil against multidrug-resistant clinical isolates. Front Microbiol 9:2329. 30344513 10.3389/fmicb.2018.02329 PMC6182053

[CIT0027] McClellan KJ, Markham A. (1999). Finasteride. A review of its use in male pattern hair loss. Drugs 57:111–126. 9951956 10.2165/00003495-199957010-00014

[CIT0028] Ocaña-Fuentes A, Arranz-Gutiérrez E, Señorans FJ, Reglero G. (2010). Supercritical fluid extraction of oregano (Origanum vulgare) essentials oils: Anti-inflammatory properties based on cytokine response on THP-1 macrophages. Food Chem Toxicol 48:1568–75. 20332013 10.1016/j.fct.2010.03.026

[CIT0029] Pan X, Han K, Peng X, et al. (2013). Nanostructed cubosomes as advanced drug delivery system. Curr Pharm Des. 19:6290–7. 23470001 10.2174/1381612811319350006

[CIT0030] Perugini P, Pavanetto F. (1998). Liposomes containing boronophenylalanine for boron neutron capture therapy. J Microencapsul 15:473–83. 9651869 10.3109/02652049809006874

[CIT0031] Safavi KH, Muller SA, Suman VJ, et al. Incidence of alopecia areata in Olmsted County, Minnesota, 1975 through 1989. Mayo Clin Proc 1995;70:628–33. 7791384 10.4065/70.7.628

[CIT0032] Salama HA, Mahmoud AA, Kamel AO, et al. (2012). Phospholipid based colloidal poloxamer-nanocubic vesicles for brain targeting via the nasal route. Colloids Surf B Biointerfaces 100:146–54. 22766291 10.1016/j.colsurfb.2012.05.010

[CIT0033] Salem HF, Nafady MM, Ewees MGE-D, et al. (2021). Rosuvastatin calcium-based novel nanocubic vesicles capped with silver nanoparticles-loaded hydrogel for wound healing management: optimization employing Box-Behnken design: in vitro and in vivo assessment. J Liposome Res 11:1–17. 10.1080/08982104.2020.1867166 33353435

[CIT0034] Shelke S, Shahi S, Jalalpure S, et al. (2016). Poloxamer 407-based intranasal thermoreversible gel of zolmitriptan-loaded nanoethosomes: formulation, optimization, evaluation and permeation studies. J Liposome Res 26:313–23. 26758957 10.3109/08982104.2015.1132232

[CIT0035] Shin SC, Cho CW, Oh IJ. (2001). Effects of non-ionic surfactants as permeation enhancers towards piroxicam from the poloxamer gel through rat skins. Int J Pharm 222:199–203. 17 11427350 10.1016/s0378-5173(01)00699-8

[CIT0036] Soleymani SM, Salimi A. (2019). Enhancement of dermal delivery of finasteride using microemulsion systems. Adv Pharm Bull 9:584–92. 31857962 10.15171/apb.2019.067 PMC6912190

[CIT0037] Songkro S, Rades T, Becket G. (2009). Effects of some terpenes on the in vitro permeation of LHRH through newborn pig skin. Die Pharmazie-An. Int. J. Pharm. Sci 64:110–5. 19320284

[CIT0038] Soylu EM, Soylu S, Kurt S. (2006). Antimicrobial activities of the essential oils of various plants against tomato late blight disease agent Phytophthora infestans. Mycopathologia 161:119–28. 16463095 10.1007/s11046-005-0206-z

[CIT0039] Wang E, Lee J-S, Hee T. (2012). Is Propionibacterium acnes associated with hair casts and alopecia? Int J Trichology 4:93–7. 23180917 10.4103/0974-7753.96907 PMC3500081

[CIT0040] Williams AC, Barry BW. (2004). Penetration enhancers. Adv Drug Deliv Rev 56:603–18. 15019749 10.1016/j.addr.2003.10.025

[CIT0041] Yan F, Azizi A, Janke S, et al. (2016). Antioxidant capacity variation in the oregano (Origanum vulgare L.) collection of the German National Genebank. Ind Crops Prod 92:19–25. Elsevier;

[CIT0042] Yan K, Sun X, Wang G, et al. (2019). Pharmacological activation of thermo-transient receptor potential vanilloid 3 channels inhibits hair growth by inducing cell death of hair follicle outer root sheath. J Pharmacol Exp Ther 370:299–307. 31152005 10.1124/jpet.119.258087

